# Lattice Boltzmann Simulation of Spatial Fractional Convection–Diffusion Equation

**DOI:** 10.3390/e26090768

**Published:** 2024-09-07

**Authors:** Xiaohua Bi, Huimin Wang

**Affiliations:** 1 School of Liberal Arts and Sciences, North China Institute of Aerospace Engineering, Langfang 065000, China; bxh_hh@126.com; 2 College of Applied Mathematics, Jilin University of Finance and Economics, Changchun 130117, China

**Keywords:** lattice Boltzmann method, spatial fractional convection–diffusion equation, numerical simulation, Riemann–Liouville fractional derivative

## Abstract

The space fractional advection–diffusion equation is a crucial type of fractional partial differential equation, widely used for its ability to more accurately describe natural phenomena. Due to the complexity of analytical approaches, this paper focuses on its numerical investigation. A lattice Boltzmann model for the spatial fractional convection–diffusion equation is developed, and an error analysis is carried out. The spatial fractional convection–diffusion equation is solved for several examples. The validity of the model is confirmed by comparing its numerical solutions with those obtained from other methods The results demonstrate that the lattice Boltzmann method is an effective tool for solving the space fractional convection–diffusion equation.

## 1. Introduction

Fractional differential equations have significant applications across various disciplines, including physics, biology, finance, and groundwater, as they better describe certain physical phenomena and material motion processes [1,2]. The rise of fractional differential equations in multiple disciplines broadens their development prospects. Due to the existence of weak singular integrals in fractional differential operators, it is very difficult to solve fractional differential equations accurately. Even if the exact solution is obtained, the exact solution is very complicated and inconvenient to use. This complexity underscores the importance of numerical studies on fractional differential equations, which are drawing growing attention. Additionally, studying fractional partial differential equations is more challenging than investigating fractional ordinary differential equations. The fractional convection–diffusion equation is an important branch of the fractional partial differential equation. It is a generalized form of the integer-order convection–diffusion equation for describing the super-diffusion phenomenon and convection–diffusion transmission. A large number of experiments show that the fractional convection–diffusion equation is more suitable for describing the Brownian motion of free particles, which has attracted wide attention [3,4,5,6,7]. At present, there have been some numerical studies on the fractional convection–diffusion equation, including the finite difference method [8,9], the finite element method [10], the spectral method, and so on [11,12]. However, in the expansive field of fractional partial differential equations, these studies are insufficient, and new research methods are needed. In this paper, the lattice Boltzmann method is used to study the Riemann–Liouville space fractional convection–diffusion equation. This equation is significant in modeling anomalous diffusion, which is commonly observed in various complex systems, such as porous media, turbulent flows, and biological tissues. The fractional derivative provides a more accurate representation of the memory and spatial heterogeneity in such systems, making it a pertinent choice for our study.

The lattice Boltzmann method is a modeling and numerical simulation method developed over the past forty years [13,14,15]. It has significant applications in the field of computational fluid dynamics and nonlinear partial differential equations [16,17,18,19,20,21,22,23,24,25,26,27]. Especially in recent years, the lattice Boltzmann method has also been applied to solve fractional differential equations, with studies extending to time fractional partial differential equations [28,29,30,31,32]. The lattice Boltzmann method, originated from lattice gas automata, is a mesoscopic numerical method. The traditional method is to discretize the macroscopic equations and solve the equations to obtain the macroscopic variables. Different from the traditional numerical method, the lattice Boltzmann method defines the macro variables by the ensemble average of the distribution function, avoiding the need to solve numerous equations. This feature makes the lattice Boltzmann method calculation simpler and has the advantages of essential parallelism, simple code, and easy handling of boundaries.

In this paper, we use the lattice Boltzmann method to solve the following Riemann–Liouville space fractional advection–diffusion equation in Equation (1).

(1) $$\frac{\partial u(x,t)}{\partial t}+b\frac{\partial u(x,t)}{\partial x}-a\frac{\partial^{\beta }u(x,t)}{\partial x^{\beta }}+du(x,t)=0,\;0<x<l,t>0,$$

where b≥0,a>0,d≥0,1<β≤2,l>0, and $\frac{\partial^{\beta }u(x,t)}{\partial x^{\beta }}$ represents the Riemann–Liouville-type fractional derivative. According to fractional derivative theory, $\frac{\partial^{\beta }u(x,t)}{\partial x^{\beta }}$ can be expressed as $\frac{\partial^{2}}{\partial x^{2}}I_{x}^{2-\beta }u(x,t)$; $\left(I_{x}^{2-\beta }u)(x,t\right)$ is Riemann–Liouville’s integral and it can be inferred that

(2) $$\left(I_{x}^{2-\beta }u\right)(x,t)=\frac{1}{\Gamma (2-\beta )}\int_{0}^{x}{\left(x-\xi \right)}^{1-\beta }u(\xi ,t)d\xi ,\;x>0,$$

Therefore, Equation (1) can be rewritten in the following form:

(3) $$\frac{\partial u(x,t)}{\partial t}+b\frac{\partial u(x,t)}{\partial x}-a\frac{\partial^{2}}{\partial x^{2}}I_{x}^{2-\beta }u(x,t)+du(x,t)=0,\;0<x<l,t>0.$$

## 2. Lattice Boltzmann Model

### 2.1. Lattice Boltzmann Equation

In this section, we construct a lattice Boltzmann model for Equation (3). The D1Q3 model is used to discretize the one-dimensional space, where $e_{\alpha }=(0,c,-c)$, $e_{\alpha }=0$ represents the velocity of stationary particles, and $\left|e_{\alpha }\right|=c$ represents the speed of moving particles. Let $f_{\alpha }(x,t)$ denote the distribution function of a single particle with velocity $e_{\alpha }$ at position x and time t; $f_{\alpha }^{eq}(x,t)$ denotes the equilibrium distribution function, which satisfies the conservation condition:

(4) $$\sum_{\alpha }f_{\alpha }^{eq}(x,t)=\sum_{\alpha }f_{\alpha }(x,t),$$

Then, $f_{\alpha }(x,t)$ evolves according to the following Equation:

(5) $$f_{\alpha }(x+e_{\alpha },t+1)-f_{\alpha }(x,t)=-\frac{1}{\tau }[f_{\alpha }(x,t)-f_{\alpha }^{eq}(x,t)]+\Omega_{\alpha }(x,t),$$

where τ is the single relaxation time and $\Omega_{\alpha }$ is an additional source term. On the physical plane, the lattice Boltzmann equation can be written as follows:

(6) $$f_{\alpha }(x+e_{\alpha }\Delta t,t+\Delta t)-f_{\alpha }(x,t)=-\frac{1}{\tau }[f_{\alpha }(x,t)-f_{\alpha }^{eq}(x,t)].$$

The Knudsen number ε is defined as $\varepsilon =\frac{l}{L}$, where l represents the mean free path of the molecule and L represents the characteristic scale of the system. Assuming that the Knudsen number ε is equal to the time step Δt, the lattice Boltzmann Equation (6) can be rewritten as follows:

(7) $$f_{\alpha }(x+\varepsilon e_{\alpha },t+\varepsilon )-f_{\alpha }(x,t)=-\frac{1}{\tau }[f_{\alpha }(x,t)-f_{\alpha }^{eq}(x,t)].$$

A Taylor expansion is performed on the left side of Equation (7)

(8) $$f_{\alpha }(x+\varepsilon e_{\alpha },t+\varepsilon )-f_{\alpha }(x,t)=\sum_{n=1}^{\infty }\frac{\varepsilon^{n}}{n!}{\left(\frac{\partial }{\partial t}+e_{\alpha }\frac{\partial }{\partial x}\right)}^{n}f_{\alpha }(x,t).$$

Retaining the item $O(\varepsilon^{4})$, we obtain

(9) $$f_{\alpha }(x+\varepsilon e_{\alpha },t+\varepsilon )-f_{\alpha }(x,t)=\sum_{n=1}^{3}\frac{\varepsilon^{n}}{n!}{\left(\frac{\partial }{\partial t}+e_{\alpha }\frac{\partial }{\partial x}\right)}^{n}f_{\alpha }(x,t)+O(\varepsilon^{4}).$$

Assuming that the Knudsen number is a small parameter, we perform a Chapman Enskog expansion on $f_{\alpha }(x,t)$ [33], yielding

(10) $$f_{\alpha }=\sum_{n=0}^{3}\varepsilon^{n}f_{\alpha }^{\left(n\right)}+O(\varepsilon^{4}),$$

where $f_{\alpha }^{\left(0\right)}=f_{\alpha }^{eq}$. Summing Equation (10) and combining it with Equation (4), we obtain

(11) $$\sum_{\alpha }f_{\alpha }^{\left(n\right)}(x,t)=0,\;n\geq 1.$$

Introducing $t_{0}$, $t_{1}$, and $t_{2}$ as different time scales, they are defined as

(12) $$\frac{\partial }{\partial t}=\sum_{n=0}^{5}\varepsilon^{n}\frac{\partial }{\partial t_{n}}+O(\varepsilon^{6}).$$

In summary, by combining Taylor expansion, multi-scale expansion, and Chapman-Enskog expansion, a series of partial differential equations at different time scales can be obtained [34]:

(13) $$C_{1}\Delta f_{\alpha }^{\left(0\right)}=-\frac{1}{\tau }f_{\alpha }^{\left(1\right)}+\Omega_{\alpha }^{\left(1\right)},$$

(14) $$\frac{\partial }{\partial t_{1}}f_{\alpha }^{\left(0\right)}+C_{2}\Delta^{2}f_{\alpha }^{\left(0\right)}+\Delta \tau \Omega_{\alpha }^{\left(1\right)}=-\frac{1}{\tau }f_{\alpha }^{\left(2\right)}+\Omega_{\alpha }^{\left(2\right)},$$

(15) $$C_{3}\Delta^{3}f_{\alpha }^{\left(0\right)}+2C_{2}\Delta \frac{\partial }{\partial t_{1}}f_{\alpha }^{\left(0\right)}+\frac{\partial }{\partial t_{2}}f_{\alpha }^{\left(0\right)}+\tau \frac{\partial }{\partial t_{1}}\Omega_{\alpha }^{\left(1\right)}+C_{2}\tau \Delta^{2}\Omega_{\alpha }^{\left(1\right)}+\tau \Delta \Omega_{\alpha }^{\left(2\right)}=-\frac{1}{\tau }f_{\alpha }^{\left(3\right)}+\Omega_{\alpha }^{\left(3\right)}.$$

Here, the partial differential operator is $\Delta \equiv \frac{\partial }{\partial t_{0}}+e_{\alpha }\frac{\partial }{\partial x}$.

Equations (16)–(18) are referred to as a series of partial differential equations at different time scales. The polynomials of the relaxation time factor τ in Equations (16)–(18) are as follows

(16) $$C_{1}=1,$$

(17) $$C_{2}=\frac{1}{2}-\tau ,$$

(18) $$C_{3}=\tau^{2}-\tau +\frac{1}{6}.$$

### 2.2. Recovery of the Macroscopic Equation

Define the macroscopic quantity u as

(19) $$u=\sum_{\alpha }f_{\alpha }(x,t).$$

According to the conservation condition,

(20) $$u=\sum_{\alpha }f_{\alpha }^{\left(0\right)}(x,t).$$

The moments of the equilibrium distribution function are

(21) $$m^{0}=\sum_{\alpha }f_{\alpha }^{\left(0\right)}e_{\alpha }=bu,$$

(22) $$\pi^{0}=\sum_{\alpha }f_{\alpha }^{\left(0\right)}e_{\alpha }^{2}=bu-\frac{a}{\varepsilon C_{2}}I_{x}^{2-\beta }u,$$

where the Riemann–Liouville integral $I_{x}^{2-\beta }u$ can be approximately calculated based on the Grünwald–Letnikov fractional derivative definition,

(23) $$I_{x}^{2-\beta }u(x,t)\approx h^{2-\beta }\sum_{r=0}^{\left[\frac{x-0}{h}\right]}\left[\begin{array}{c} 2-\beta \\ r \end{array}\right]u(x-rh,t),\;0<x<l,$$

where $\left[\begin{array}{c} 2-\beta \\ r \end{array}\right]=\frac{\left(2-\beta )(2-\beta +1)\cdots (2-\beta +r-1\right)}{r!}$, for r>0, and $\left[\begin{array}{c} 2-\beta \\ r \end{array}\right]=1$, for r=0.

Assuming that $\Omega_{\alpha }=\varepsilon^{2}\Omega_{\alpha }^{\left(2\right)}$, i.e., $\Omega_{\alpha }^{\left(n\right)}=0$, n≠2, then from $\sum_{\alpha }\left[\left(13)+\varepsilon \times (14\right)\right]$, it can be concluded that

(24) $$\frac{\partial u(x,t)}{\partial t}+b\frac{\partial u(x,t)}{\partial x}-a\frac{\partial^{2}}{\partial x^{2}}I_{x}^{2-\beta }u(x,t)=\varepsilon \sum_{\alpha }\Omega_{\alpha }^{\left(2\right)}+O(\varepsilon^{2}),\;0<x<l,t>0.$$

Equation (24) is an approximate expression for the recovered macroscopic Equation (1). Assuming that $\Omega_{\alpha }^{\left(2\right)}$ is independent of α, then

(25) $$\Omega_{\alpha }^{\left(2\right)}=\Omega^{\left(2\right)}=\frac{-du}{3\varepsilon }$$

By combining the D1Q3 model and solving Equations (20)–(22), the equilibrium distribution function is obtained

(26) $$f_{\alpha }^{\left(0\right)}=\left\{\begin{array}{c} \frac{1}{2c^{2}}(bu-\frac{a}{\varepsilon C_{2}}I_{x}^{2-\beta }u)+\frac{bu}{2c},\;\alpha =1,2,\\ \frac{1}{2c^{2}}(bu-\frac{a}{\varepsilon C_{2}}I_{x}^{2-\beta }u)-\frac{bu}{2c},\;\alpha =1,2,\\ u-f_{1}^{\left(0\right)}-f_{2}^{\left(0\right)},\;\alpha =0. \end{array}\right.$$

Summing $\left(13)+\varepsilon \times (14)+\varepsilon^{2}\times (15\right)$ over α yields

(27) $$\frac{\partial u(x,t)}{\partial t}+b\frac{\partial u(x,t)}{\partial x}-a\frac{\partial^{2}}{\partial x^{2}}I_{x}^{2-\beta }u(x,t)=-du(x,t)+E_{2}+O(\varepsilon^{2}),$$

The error analysis shows that the error term is

(28) $$\begin{array}{l} E_{2}=\varepsilon^{2}\left(C_{3}\sum_{\alpha }\Delta^{3}f_{\alpha }^{\sigma ,(0)}+2C_{2}\sum_{\alpha }\Delta \frac{\partial }{\partial t_{1}}f_{\alpha }^{\sigma ,(0)}+\tau \sum_{\alpha }\Delta \Omega_{\alpha }^{\sigma ,(2)}\right)\\ \;=-\varepsilon^{2}C_{3}\left[(bc^{2}-b^{3})\frac{\partial^{3}u}{\partial x^{3}}+(2+\frac{1}{\varepsilon C_{2}})ab\frac{\partial^{1+\beta }u}{\partial x^{1+\beta }}\right]-\varepsilon \tau db\frac{\partial u}{\partial x} \end{array},$$

Thus, the macroscopic Equation (1) is recovered as

(29) $$\frac{\partial u(x,t)}{\partial t}+b\frac{\partial u(x,t)}{\partial x}-a\frac{\partial^{\beta }u(x,t)}{\partial x^{\beta }}+du(x,t)=O(\varepsilon ).$$

## 3. Numerical Example

Several examples are provided to demonstrate the numerical solution of the equation using the constructed model.

**Example** **1.** *In this example,* b=1.0,a=1.0,d=1.0*. The initial and boundary conditions are specified as follows:*

(30) u(x,0)=x, 0<x<1,

(31) $$u(0,t)=0,\;\frac{\partial u(1,t)}{\partial x}=0.$$

**Figure 1:**
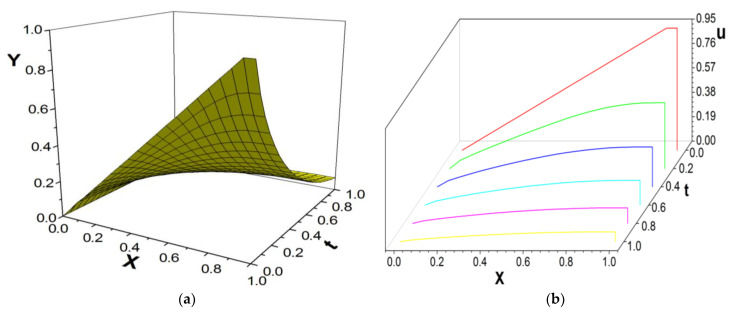
LBM solution, from t=0 to t=1.0; (**a**) the propagation of solution; (**b**) waterfall plot; parameters are M=21,Δt=0.005, Δx=0.05, c=Δx/Δt, τ=1.095, b=1.0,a=1.0,d=1.0, and β=1.5.

**Figure 2:**
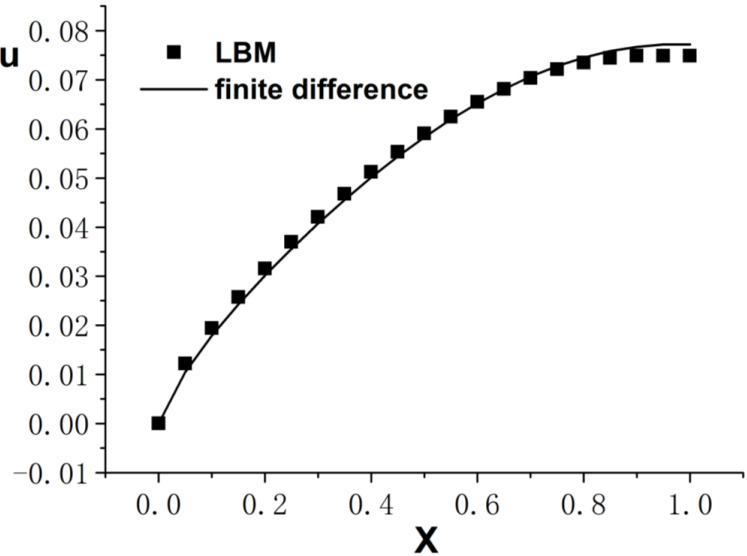
Comparison of LBM solution and finite difference solution; parameters are M=21,Δt=0.005, Δx=0.05, c=Δx/Δt, τ=1.095, t=1.0, b=1.0, a=1.0, d=1.0, β=1.5.

**Figure 3:**
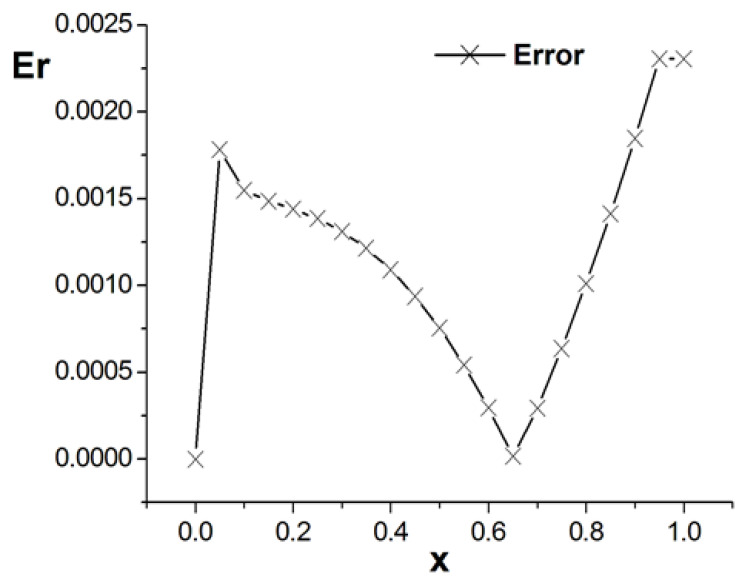
Error curve; parameters are M=21,Δt=0.005, Δx=0.05, c=Δx/Δt, τ=1.095, t=1.0, b=1.0,a=1.0,d=1.0, and β=1.5.

Figure 1, Figure 2 and Figure 3 present the numerical results with β=1.5, with the computational parameters M=21,Δt=0.005, Δx=0.05, c=Δx/Δt, and τ=1.095. Figure 1 shows the LBM solution from t=0 to t=1.To verify the model’s validity, we use the finite difference scheme as a reference and compare its solution with the LBM solution [35]. Figure 2 compares the LBM solution with the finite difference solution. Figure 3 presents the error curve $Er=\left|u^{L}-u^{D}\right|$ at t=1, where $u^{L}$ represents the LBM solution and $u^{D}$ denotes the finite difference solution. The results indicate that the LBM solution is in good agreement with the finite difference solution.

**Figure 4:**
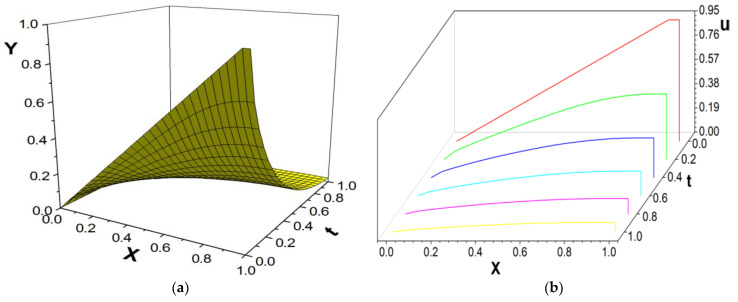
LBM solution, from t=0 to t=1.0, (**a**) the propagation of solution; (**b**) waterfall plot; parameters are M=31,Δt=0.001, Δx=1.0/(M−1), c=Δx/Δt, τ=1.23, b=1.0,a=1.0,d=1.0, β=1.8.

**Figure 5:**
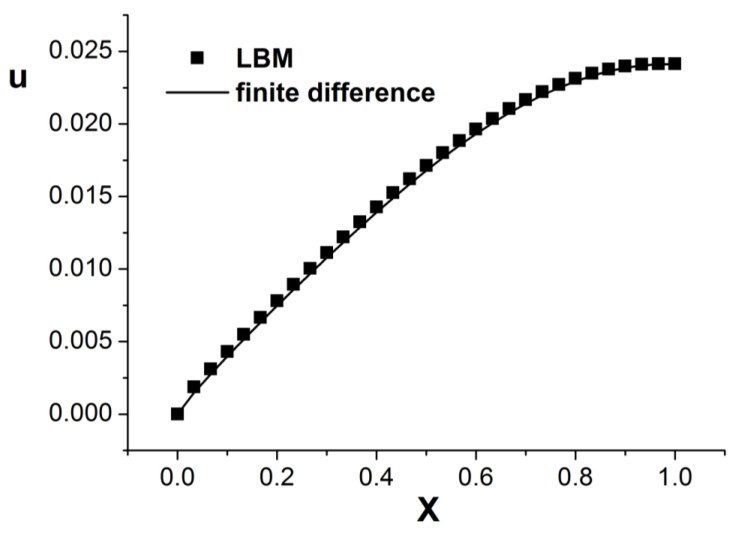
Comparison of LBM solution and finite difference solution, parameters are M=31,Δt=0.001, Δx=1.0/(M−1), c=Δx/Δt, τ=1.23, t=1.0, b=1.0,a=1.0, d=1.0, and β=1.8.

**Figure 6:**
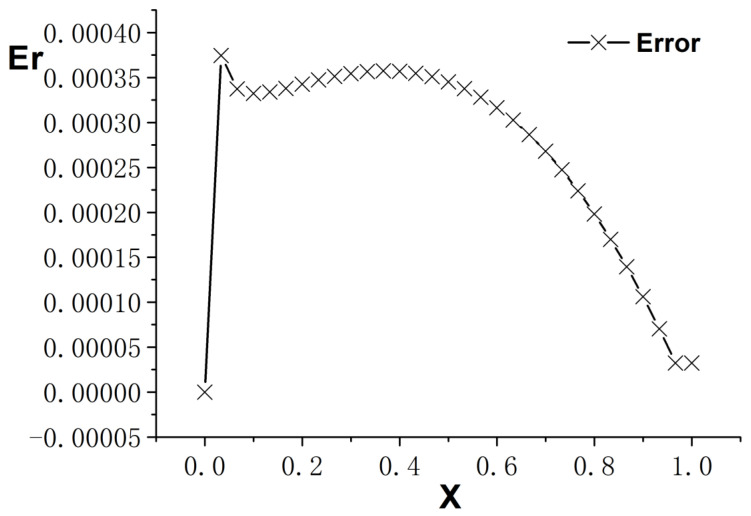
Error curve; parameters are Δt=0.001, Δx=1.0/(M−1), c=Δx/Δt, τ=1.23, t=1.0, b=1.0,a=1.0,d=1.0, and β=1.8.

**Figure 7:**
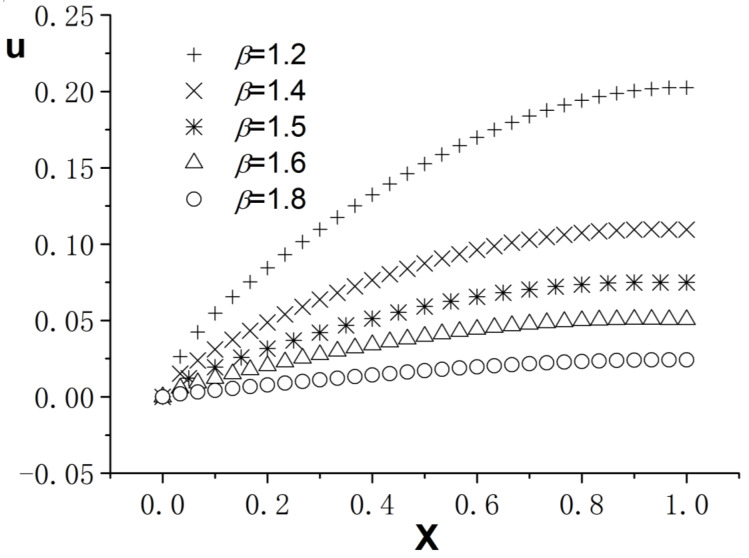
LBM solutions with different parameter β; b=1.0,a=1.0, d=1.0, t=1.0.

Figure 4, Figure 5 and Figure 6 present the numerical results with β=1.8; the computational parameters are the number of lattices M=31,Δt=0.001, Δx=1.0/(M−1), c=Δx/Δt, and τ=1.23. Figure 4 gives the LBM solution from t=0 to t=1. Figure 5 compares the LBM solution with the finite difference solution. Figure 6 presents the error curve $Er=\left|u^{L}-u^{D}\right|$ at t=1, where $u^{L}$ denotes the LBM solution and $u^{D}$ represents the finite difference solution. Figure 7 shows the LBM solution with varying parameter β.

**Example** **2.** *In this example,* b=1.0,a=1.0,d=0*. The initial and boundary conditions are specified as follows:*

(32) u(x,0)=x,0<x<1.

(33) $$u(0,t)=0,\frac{\partial u(1,t)}{\partial x}=0.$$

**Figure 8:**
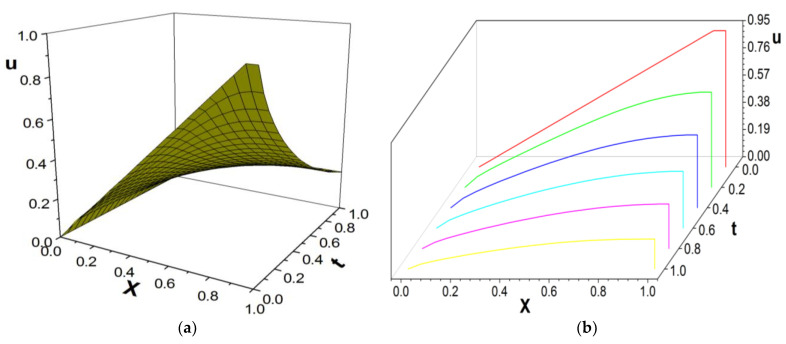
LBM solution, t=0 to t=1.0; (**a**) the propagation of solution; (**b**) waterfall plot; parameters are M=21,Δt=0.005, Δx=0.05, c=Δx/Δt, τ=1.07, b=1.0,a=1.0,d=0, and β=1.5.

**Figure 9:**
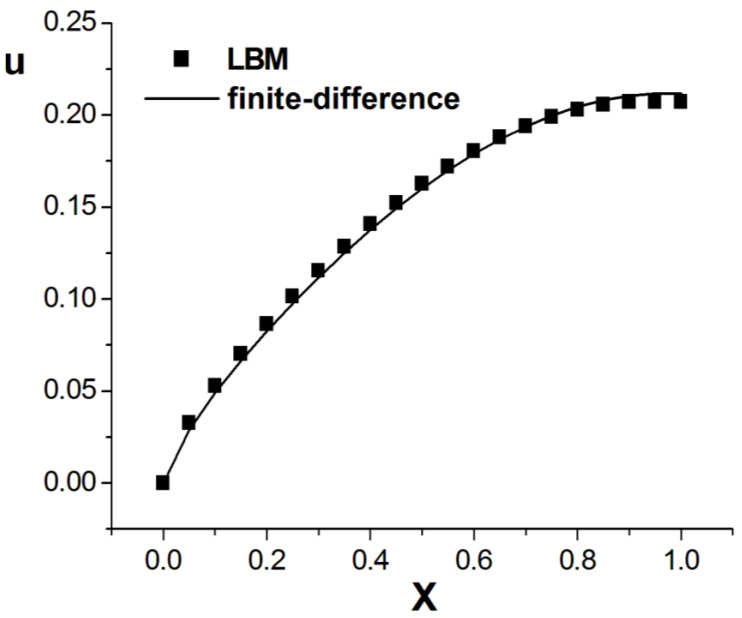
Comparison of LBM solution and finite difference solution; parameters are M=21,Δt=0.005, Δx=0.05, c=Δx/Δt, τ=1.07, t=1.0, b=1.0,a=1.0,d=0, and β=1.5.

**Figure 10:**
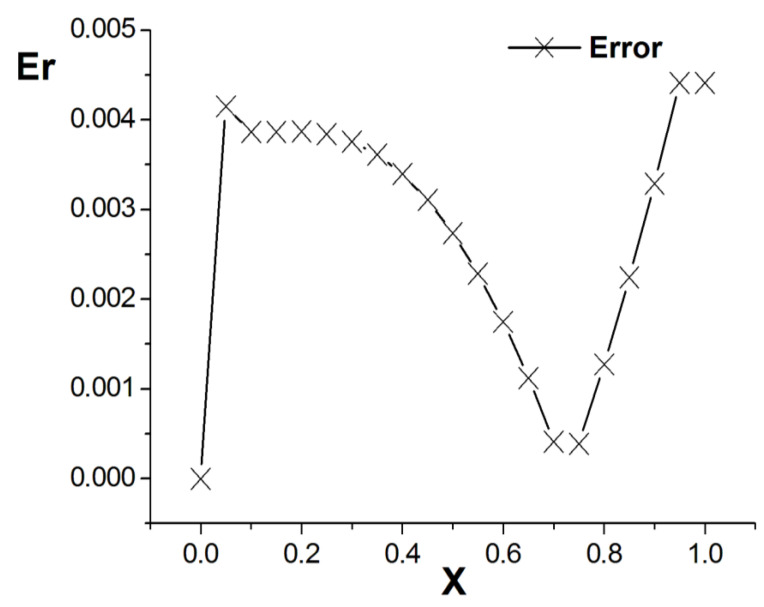
Error curve; parameters are M=21,Δt=0.005, Δx=0.05, c=Δx/Δt, τ=1.07, t=1.0, b=1.0,a=1.0,d=0, and β=1.5.

Figure 8, Figure 9 and Figure 10 present the numerical results with β=1.5; the computational parameters are M=21,Δt=0.005, Δx=0.05, c=Δx/Δt, and τ=1.07. Figure 8 shows the LBM solution from t=0 to t=1. Figure 9 compares the LBM solution with the finite difference solution [35]. Figure 10 shows the error curve $Er=\left|u^{L}-u^{D}\right|$ at t=1, where $u^{L}$ represents the LBM solution and $u^{D}$ represents the finite difference solution. The results show that the LBM solution agrees with the finite difference solution.

**Figure 11:**
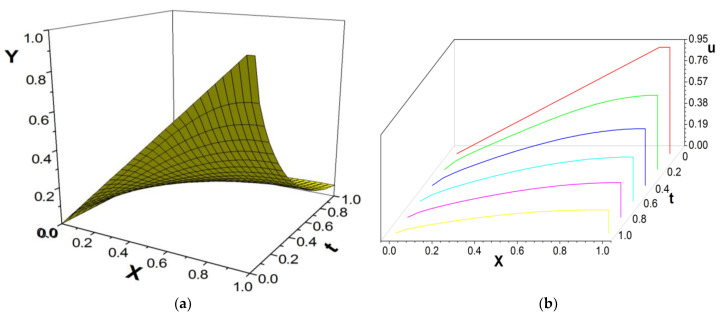
LBM solution, t=0 to t=1.0; (**a**) the propagation of solution; (**b**) waterfall plot; parameters are M=31,Δt=0.001, Δx=1.0/(M−1), c=Δx/Δt, τ=1.17, b=1.0,a=1.0,d=0, and β=1.8.

**Figure 12:**
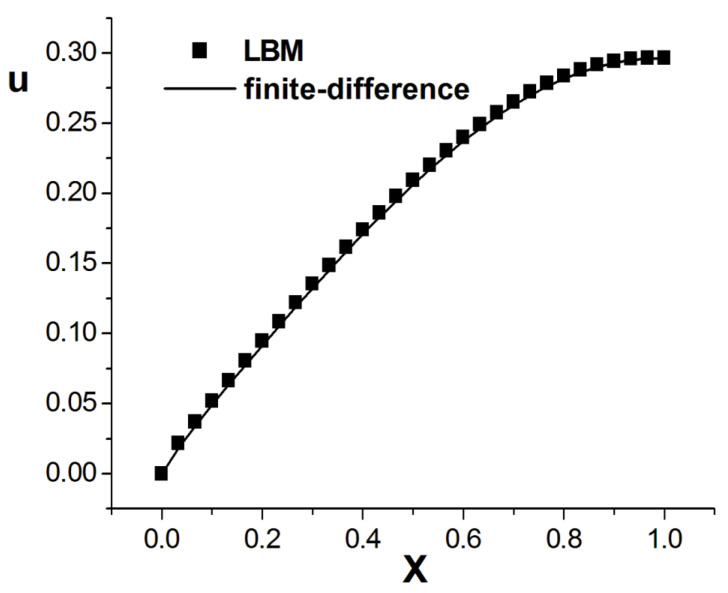
Comparison of LBM solution and finite difference solution; parameters are M=31,Δt=0.001, Δx=1.0/(M−1), c=Δx/Δt, τ=1.17, t=0.4, b=1.0,a=1.0,d=0, and β=1.8.

**Figure 13:**
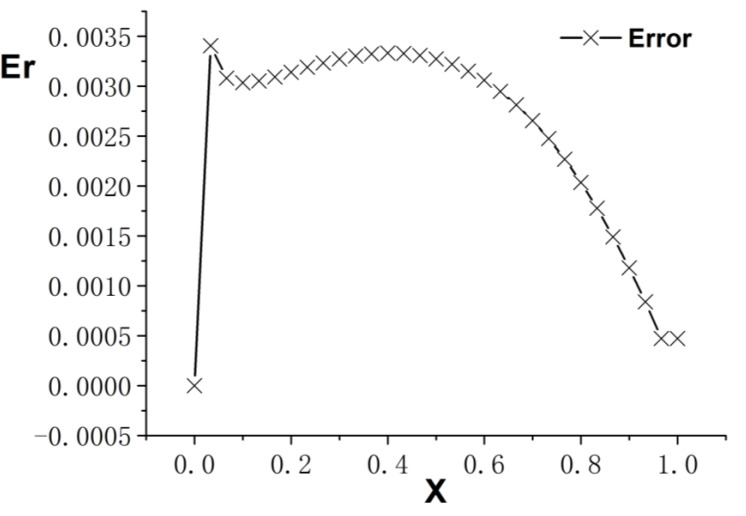
Error curve; parameters are M=31,Δt=0.001, Δx=1.0/(M−1), c=Δx/Δt, τ=1.17,t=0.4, b=1.0,a=1.0,d=0, and β=1.8.

**Figure 14:**
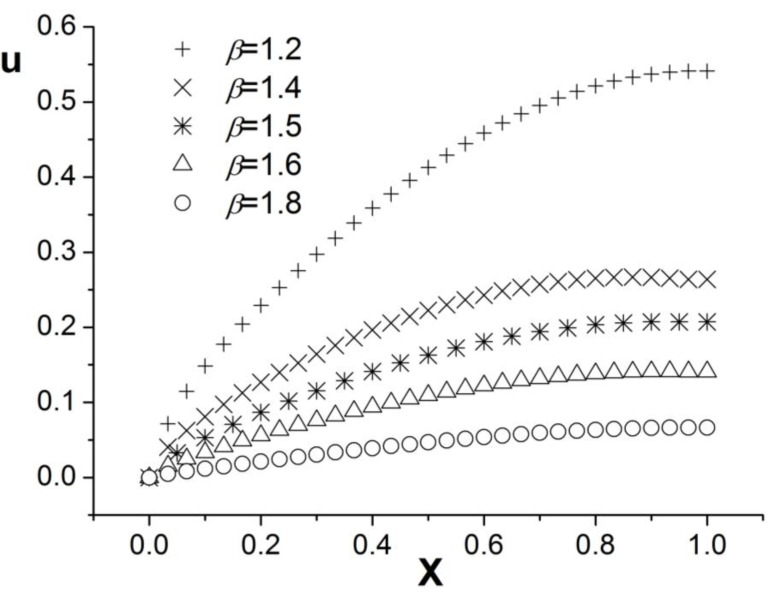
Comparison of LBM solutions with different parameter β; b=1.0,a=1.0, d=0, t=1.0.

Figure 11 are the numerical results with β=1.8; the computational parameters are the number of lattices M=31,Δt=0.001, Δx=1.0/(M−1), c=Δx/Δt, and τ=1.17. Figure 11 gives the LBM solution at t=0 to t=1. Figure 12 shows the LBM solution at t=0.4 against the finite difference solution [35]. Figure 13 shows the error curve $Er=\left|u^{L}-u^{D}\right|$ at t=0.4, where $u^{L}$ represents the LBM solution and $u^{D}$ represents the finite difference solution. Figure 14 shows the LBM solution with different parameter β.

## 4. Conclusions

This paper presents a numerical study of the Riemann–Liouville space fractional convection–diffusion equation and proposes a lattice Boltzmann model for its solution. Taylor expansion, time multi-scale expansion, and Chapman expansion of the lattice Boltzmann equation are conducted to derive a series of partial differential equations across different time scales. Macro variables are defined through the moments of the distribution function, and the macro equations are obtained by reconstructing the series of partial differential equations. The lattice Boltzmann model is then applied to solve the space fractional convection–diffusion equation, demonstrated through several examples. The lattice Boltzmann solution is compared with the finite difference scheme to verify the effectiveness of the proposed model. The numerical results indicate that the lattice Boltzmann method effectively solves the Riemann–Liouville space fractional convection–diffusion equation. This approach provides valuable insights for solving other spatial fractional partial differential equations and holds significant potential for advancing the field of fractional partial differential equations. We will consider extending this model to two-dimensional and three-dimensional space fractional convection–diffusion equations, but there are still some challenges that need to be addressed, which we will study in future work.

## Data Availability

The original contributions presented in the study are included in the article, further inquiries can be directed to the corresponding author/s.
